# Long-term stability of anti-cyclic citrullinated peptide antibody status in patients with early inflammatory polyarthritis

**DOI:** 10.1186/ar3834

**Published:** 2012-05-09

**Authors:** Marian L Burr, Sebastien Viatte, Marwan Bukhari, Darren Plant, Deborah P Symmons, Wendy Thomson, Anne Barton

**Affiliations:** 1 Arthritis Research UK Epidemiology Unit, Manchester Academy of Health Sciences, The University of Manchester, Stopford Building, Oxford Road, Manchester M13 9PT, UK; 2 Cambridge Institute for Medical Research, Wellcome Trust/MRC Building, University of Cambridge, Cambridge CB2 0XY, UK

## Abstract

**Introduction:**

The utility of reassessing anti-cyclic citrullinated peptide (anti-CCP) antibody status later in disease in patients presenting with early undifferentiated inflammatory polyarthritis, particularly in those who test negative for both anti-CCP and rheumatoid factor (RF) at baseline, remains unclear. We aimed therefore to determine the stability of CCP antibody status over time and the prognostic utility of repeated testing in subjects with early inflammatory polyarthritis (IP).

**Methods:**

Anti-CCP and RF were measured at baseline and 5 years in 640 IP patients from the Norfolk Arthritis Register, a primary care-based inception cohort. The relation between change in anti-CCP status/titer and the presence of radiologic erosions, the extent of the Larsen score, and Health Assessment Questionnaire (HAQ) score by 5 years was investigated.

**Results:**

With a cut-off of 5 U/ml, 28% subjects tested positive for anti-CCP antibodies, 29% for RF, and 21% for both at baseline. Nine (2%) anti-CCP-negative patients seroconverted to positive, and nine (4.6%) anti-CCP-positive individuals became negative between baseline and 5 years. In contrast, RF status changed in 17% of subjects. However, change in RF status was strongly linked to baseline anti-CCP status and was not independently associated with outcome. Ever positivity for anti-CCP antibodies by 5 years did not improve prediction of radiographic damage compared with baseline status alone (accuracy, 75% versus 74%). A higher baseline anti-CCP titer (but not change in anti-CCP titer) predicted worse radiologic damage at 5 years (*P *< 0.0001), even at levels below the cut-off for anti-CCP positivity. Thus, a titer of 2 to 5 U/ml was strongly associated with erosions by 5 years (odds ratio, 3.6 (1.5 to 8.3); *P *= 0.003).

**Conclusions:**

Repeated testing of anti-CCP antibodies or RF in patients with IP does not improve prognostic value and should not be recommended in routine clinical practice.

## Introduction

The management of rheumatoid arthritis (RA) has undergone a seismic shift in recent years, with early intensive intervention becoming the bench-mark. Diagnosing RA in the early stages of its evolution may, however, be challenging. The use of biomarkers to distinguish those patients with inflammatory polyarthritis (IP) who will progress rapidly from those who will follow a more benign course is therefore of prime importance [1]. In relation to this, the presence of anti-CCP antibodies has been found to be a highly specific diagnostic marker for RA and a powerful predictor of more severe disease, worse radiological and functional outcomes and poorer response to treatment [2-9]. However, the utility of retesting anti-CCP antibodies later in disease in patients presenting with early undifferentiated IP, particularly in those who test negative for both anti-CCP and rheumatoid factor (RF) at baseline, remains unclear. Most studies addressing the prognostic role of anti-CCP antibodies in IP have relied on baseline testing alone, and evidence regarding the value of repeated testing is lacking [2,3,6]. Nevertheless, it is not uncommon in clinical practice for both anti-CCP and RF to be tested on multiple occasions in an individual patient [10].

To answer this question, two key aspects must be addressed. First, what is the likelihood that anti-CCP antibody levels will change over the course of disease in patients with IP? Second, does a change in anti-CCP antibody status or titer associate with disease-severity outcomes and, if so, would a lower threshold improve prediction of adverse outcomes?

The appearance of anti-CCP antibodies may predate the onset of RA, and anti-CCP titers have been shown to increase in the years preceding diagnosis [11-13]. A lower threshold for anti-CCP positivity may therefore be more sensitive in predicting future RA development [13]. The proportion of patients testing positive for RF or anti-CCP tend to be much lower in cohorts with early IP than in populations with established RA. However, data on serial autoantibody titers and rates of seroconversion over time in subjects with early IP are extremely scarce [14]. In a cohort of 117 patients with early arthritis, a nonsignificant increase was found in the proportion of patients positive for anti-CCP and in anti-CCP titers over a mean follow-up period of 32 months [15]; whereas in a larger IP cohort of 545 patients, anti-CCP titers decreased significantly between baseline and 1 year [16]. A recent systematic literature review concluded that, although autoantibody seroconversion appears to occur relatively infrequently, further studies are needed to determine both the likelihood and prognostic implications of anti-CCP or RF seroconversion in subjects with undifferentiated IP [14].

Higher anti-CCP titers have been shown to predict more-rapid radiographic progression in RA [5,17]. However, the prognostic importance of anti-CCP titer in undifferentiated IP has not been established. Furthermore, it is not clear how change in anti-CCP titer over time relates to treatment or outcomes [17-19]. In patients with RA, one study found no correlation between change in anti-CCP levels and changes in clinical indices, whereas, in another, increasing anti-CCP titers were associated with greater radiographic progression [17,18]. A study evaluating this relation in subjects with IP found no significant association between first-year change in anti-CCP titer and outcomes at 2 years [16].

The aims of this study therefore were to assess the long-term stability of anti-CCP antibody status in patients with IP and to evaluate the relation between anti-CCP titer and radiographic and functional outcome to determine whether repeated antibody testing adds any additional prognostic value.

## Materials and methods

### Patients

Subjects were recruited by the Norfolk Arthritis Register (NOAR), a primary care-based inception cohort of patients with IP, between 1990 and 1994. Details of NOAR have been published previously [20]. In brief, adults aged ≥ 16 years with swelling of two or more joints lasting 4 weeks or longer were referred to NOAR. Those subsequently diagnosed with a condition other than RA, IP, psoriatic arthritis, or postviral arthritis were excluded. Subjects were assessed by a trained metrologist within 2 weeks by using a standardized approach and then annually for 3 years and again at the fifth year. Serum analysis for RF, CRP, and anti-CCP was performed on blood samples taken at baseline and 5 years. Data gathered at baseline and year 5 included joint counts for swelling and tenderness (in 51 joints) and a patient-completed Health Assessment Questionnaire (HAQ). DAS28 was calculated by using CRP levels and 28 tender- and swollen-joint counts (online at [21]). Detailed information on the use of disease-modifying anti-rheumatic drugs (DMARDS) and steroids was collected. Radiographs of the hands and feet were performed at 5 years and scored by using the Larsen technique [22]. All radiographs were scored by two observers with a third observer arbitrating in case of disagreement [23]. The 1987 ACR classification criteria for RA were applied at baseline and cumulatively at each assessment. A blood sample for DNA extraction was obtained at baseline; *HLA DRB1 *genotyping was undertaken, and shared epitope (SE) defined, as described previously [24]. Written consent was obtained from all patients, and the study was approved by the Norwich Local Research Ethics Committee.

### Serum testing

RF was measured by using a latex method, and a titer of > 1:40 was classed as positive. CRP was measured by using rate nephelometry. Anti-CCP testing was performed by using the Axis-Shield DIASTAT kit according to manufacturer's instructions (Axis-Shield, Dundee, U.K.) by using the recommended cut-off of > 5 U/ml as positive. If the initial test gave an indeterminate value close to the cut-off point, defined as a titer between 2 and 9 U/ml, the test was repeated, and the mean of the two values taken. The assay is reliable up to concentrations of 100 U/ml, and therefore, all values above this were analyzed as 101 U/ml.

### Details of anti-CCP testing

Subjects whose anti-CCP status changed between baseline and 5 years were identified, and both baseline and 5-year samples were retested to minimize measurement error. In the event of a discrepancy between the original and repeated results, the assay was repeated a further 2 times, and the mean of the measurements was taken.

### Statistical analysis

Subjects were grouped according to stability of anti-CCP or RF status between baseline and 5 years. The following groups were defined for anti-CCP antibody status: CCP^-/-^; CCP^-/+^; CCP^+/-^; CCP^+/+^, where the first sign (+ or -) indicates status at baseline, and the second, the status by 5 years; similar groups were defined for RF status.

The groups were compared first for differences in baseline characteristics by using either the χ^2 ^test or the Fisher Exact test for dichotomous variables and the Mann-Whitney *U *test for continuous variables. Second, associations between the different autoantibody groups and outcome by 5 years (presence of erosions, presence of severe radiologic damage (highest tertile of Larsen score, > 15), functional disability (HAQ score ≥ 1), and indicators of ongoing disease activity, namely swollen and tender joint count and DAS28)) were tested by using logistic regression for binary outcomes, negative binomial regression for counts, and median regression for continuous non-normal outcomes. In the group which was autoantibody negative at baseline (CCP^-^/RF^-^), the subgroup that became autoantibody positive (either anti-CCP and/or RF positive) by 5 years was compared with those which remained negative to determine whether differences existed in 5-year outcomes. Continuous variables were compared by using the Mann-Whitney *U *test, and categoric variables were compared by using the Fisher Exact test.

Baseline anti-CCP titer was also evaluated as a continuous variable for prediction of erosions and a Larsen score > 15 by 5 years by using a receiver-operating characteristic (ROC) curve method. Where subjects were split into four categories of anti-CCP titer (≤ 2, > 2 to 5, > 5 to 20, and > 20 U/ml), logistic regression models were used to evaluate association with radiologic outcomes.

The Wilcoxon signed-rank test was used to investigate changes in anti-CCP titer in individual patients. Correlation between change in anti-CCP titers and both absolute values and change in clinical variables was assessed by using the Spearman rank correlation coefficient.

All analyses were undertaken by using Stata version 10.0, and *P *values of less than 0.05 were considered significant.

## Results

### Baseline cohort characteristics

Between 1990 and 1994, 1,098 subjects were recruited to NOAR. Included in this analysis were 640 (58%) with available sera for RF and anti-CCP testing at both baseline and 5 years. No significant differences between those included and excluded were detected for any of the baseline variables studied (data not shown). The 313 (49%) subjects fulfilled 1987 ACR criteria for RA at baseline and 463 (72%) fulfilled them cumulatively over the first 5 years of follow-up. Of the subjects, 25% had received steroids by year 5, and 47% had been treated with a DMARD. The DMARDs most commonly used were sulfasalazine in 37% of patients, methotrexate in 22%, and gold in 7%. No subjects had received a biologic agent by 5 years of follow-up, consistent with the time period in which the samples were collected.

At baseline, 46 (7%) subjects were positive for RF alone, 59 (9%), positive for anti-CCP alone, and 136 (21%), positive for both antibodies. The overall distribution of autoantibody status was similar at 5 years; 42 (7%) were positive for RF alone, 71 (11%), positive for anti-CCP alone, and 124 (19%), positive for both.

### Characteristics and 5-years outcome of patients with IP who will never satisfy 1987 criteria for RA within the first 5 years of follow-up

At baseline, patients who will satisfy cumulatively 1987 ACR criteria for rheumatoid arthritis at any time point during the first 5 years of follow-up differ significantly from those who will never satisfy those criteria within the same follow-up time (Table 1). The proportion of anti-CCP-positive patients, the overall disease severity at baseline and at 5 years, and the probability of treatment are highly significantly higher in patients who will turn out to have RA. Only eight patients of 177 (4.5%) with IP who will not be classified as RA ("non-RA inflammatory polyarthritis") are positive for anti-CCP at baseline. After 5 years, only two of those patients have seroconverted (Additional file 1, Supplemental table S4): one from anti-CCP positive to negative, and one from anti-CCP negative to positive. Such a low number of anti-CCP-positive patients prevents any powerful analysis of the differences between seronegative and seropositive non-RA inflammatory polyarthritis (Additional file 1, Supplemental table S4). For that reason, and because it is not possible for practitioners at baseline to determine with high accuracy which patient will eventually be classified as RA within the first 5 years of follow-up, we performed the rest of the analysis presented here on the entire cohort of 640 patients, without stratification by the classification at 5 years.

**Table 1:** Baseline characteristics and 5-year outcome of patients who will satisfy cumulatively 1987 ACR criteria for rheumatoid arthritis at any time during the first 5 years of follow-up, versus those who will not

Classification	Satisfying 1987 ACR criteria within the first 5 years (*n *= 463; 72%)	NEVER satisfying 1987 ACR criteria within the first 5 years (*n *= 177; 28%)	*P *value
**Baseline characteristics**			
Age (onset)	55.4 (45.8, 65.4)	47.7 (34.3, 57.9)	3.6E-09
Female	305 (66%)	114 (64%)	0.73
Symptom duration	5.3 (2.6, 11.3)	4.5 (1.9, 9.0)	0.01
Anti-CCP +ve	187 (40.4%)	8 (4.5%)	1.2E-18
Anti-CCP titer	1.6 (0.7, 42.5)	0.8 (0.5, 1.4)	6.4E-16
HAQ	0.875 (0.375, 1.5)	0.25 (0, 0.625)	5.8E-24
DAS28	4.4 (3.5, 5.4)	2.8 (2.1, 3.6)	5.2E-33
**5-year outcome**			
HAQ	1.125 (0.375, 1.75)	0.25 (0, 0.625)	4.1E-24
DAS28	2.8 (2.0, 3.9)	2.0 (1.15, 2.4)	1.3E-14
Erosions	220 (56.9%)	12 (8.8%)	3.1E-22
Larsen score	12 (2, 31)	1 (0, 4)	9.3E-21
DMARD received	278 (60.0%)	23 (13.1%)	2.2E-26

Continuous variables expressed as median (interquartile range) and compared by using the Mann-Whitney *U *test. Categoric variables expressed as number (percentage) and compared by using the χ^2 ^test. ACR, American College of Rheumatology criteria for rheumatoid arthritis; DAS, disease activity score; HAQ, Health Assessment Questionnaire.

### Anti-CCP antibody status remains stable after up to 5 years of disease in patients with IP

Anti-CCP antibody status remained remarkably stable between baseline and 5 years. Only nine (2%) anti-CCP-negative patients seroconverted to positive, and nine (4.6%) anti-CCP-positive individuals became negative (Table 2). Several of these patients had anti-CCP titers close to the cut-off point of 5. The small number of subjects in whom anti-CCP status changed limited the power to perform reliable statistical comparisons with those patients whose anti-CCP status remained stable. Nevertheless, individuals converting from negative to positive were more likely to be RF positive at baseline, to have a higher baseline anti-CCP titer, and to possess at least one copy of the shared epitope (SE) than did those who remained negative (Table 2).

**Table 2:** Baseline characteristics and 5-year outcome according to baseline and 5-year anti-CCP status

	**Anti**-**CCP negative at baseline**			**Anti**-**CCP positive at baseline**		
	
**5**-**year anti**-**CCP status**	**Anti**-**CCP negative** (*n *= 436, 98%) CCP^-/-^	Anti-CCP positive (*n *= 9, 2%) CCP^-/+^	*P *value	Anti-CCP negative (*n *= 9, 4.6%) CCP^+/-^	Anti-CCP positive (*n *= 186, 95.4%) CCP^+/+^	*P *value
**Baseline characteristic**						
Age (*n *= 640)	51.9 (40.7, 63.1)	47.1 (42.8, 57.1)	0.39	53.4 (50.1, 58.5)	56.1 (48.1, 65.0)	0.46
Female (*n *= 640)	293 (67%)	7 (78%)	0.72	5 (56%)	114 (61%)	0.74
Symptom duration (*n *= 596)	4.9 (2.1, 10.4)	3.6 (2.5, 10.1)	0.77	4.8 (3.1, 6.1)	5.3 (2.9, 10.9)	0.82
RF +ve (*n *= 640)	43 (9.8%)	3 (33%)	0.06	6 (67%)	130 (70%)	1.0
Anti-CCP titer (*n *= 596)	0.8 (0.5, 1.4)	1.5 (1.4, 3.8)	0.0004	16.2 (13.0, 41.6)	66.8 (32, 101)	0.005
SE - 1 or 2 copies (*n *= 593)	213 (53%)	7 (100%)	0.017	7 (88%)	138 (78%)	1.0
HAQ (*n *= 639)	0.6 (0.3, 1.1)	1.1 (0.6, 1.3)	0.13	0.8 (0.4, 1.5)	0.9 (0.4, 1.6)	0.88
CRP (*n *= 591)	3 (0, 11)	4 (1, 14)	0.73	10 (6, 16)	11 (4, 26)	0.83
Swollen joints (*n *= 640)	6 (2, 12)	11 (5, 13)	0.12	5 (2, 10)	8 (4, 17)	0.22
Tender joints (*n *= 640)	7 (2, 16)	15 (6, 24)	0.11	7 (5, 8)	9 (4, 16)	0.24
DAS28 (*n *= 591)	3.7 (2.7, 4.8)	5.1 (3.2, 5.5)	0.22	4.0 (3.2, 4.5)	4.5 (3.5, 5.4)	0.30
Current smoker (*n *= 640)	102 (23%)	2 (22%)	1.0	0 (0)	56 (30%)	0.06
Fulfils ACR criteria (*n *= 640)	168 (39%)	4 (44%)	0.74	6 (67%)	135 (73%)	0.71
**Outcome year 5**						
Erosions (*n *= 523)	95 (27%)	6 (85%)	0.002	6 (75%)	125 (80%)	0.66
Larsen score (*n *= 521)	2 (0, 10)	35 (19, 71)	0.001	28 (5, 44)	28 (11, 43)	0.80
HAQ (*n *= 639)	0.6 (0, 1.4)	1.6 (0.1, 2.1)	0.24	1.1 (0.4, 1.8)	1.1 (0.5, 2)	0.40
Swollen joints (*n *= 475)	1 (0, 3)	2 (1, 5)	0.07	1 (0, 2)	3 (0, 8)	0.04
Tender joints (*n *= 475)	1 (0, 6)	3 (0, 11)	0.67	2 (0, 4)	3(0, 11)	0.20
DAS28 (*n *= 463)	2.2 (1.4, 3.1)	2.9 (1.6, 4.2)	0.25	1.7 (1.2, 3.4)	3.3 (2.4, 4.4)	0.03
DMARD received (*n *= 639)	124 (29%)	8 (89%)	< 0.0001	7 (78%)	162 (87%)	0.34
Fulfils ACR criteria (*n *= 639)	268 (62%)	8 (89%)	0.16	8 (89%)	179 (96%)	0.32

Continuous variables expressed as median (interquartile range) and compared by using the Mann-Whitney *U *test. Categoric variables expressed as number (percentage) and compared by using the Fisher Exact test. ACR, American College of Rheumatology criteria for rheumatoid arthritis; CRP, C-reactive protein; DAS28, disease activity score in 28 joints; DMARD, disease-modifying antirheumatic drug;

HAQ, Health Assessment Questionnaire; RF, rheumatoid factor.

### RF status is more likely to change, and change is predicted by the baseline anti-CCP-antibody status

RF status was more likely to change between baseline and 5 years than was anti-CCP status (Additional file 1, Supplemental table S1). 48 (10%) baseline RF-negative patients tested positive at 5 years, and 64 (35%) of those initially positive tested negative at 5 years. The most notable factor associated with a change in RF status in the first 5 years of disease was baseline anti-CCP status (Additional file 1, Supplemental table S1). RF status was 8 times more likely to change in patients with a discordant baseline autoantibody status (RF^+^/anti-CCP^- ^or RF^-^/anti-CCP^+^) than in those who were either negative or positive for both auto-antibodies (odds ratio (OR) 8.2; 95% CI, 4.9 to 13.7; *P *< 0.00001). Thus, RF-negative individuals who seroconverted to RF positive were more likely to be anti-CCP positive at baseline than were those who remained RF negative (OR, 7.6; 95% CI, 3.8 to 15.3); similarly, those who converted from RF positive to RF negative were more likely to be anti-CCP negative at baseline than were those who remained RF positive (OR, 7.4; 95% CI, 3.5 to 15.6, (Additional file 1, Supplemental table S1). These associations remained significant after adjustment for other baseline variables and DMARD treatment.

### Baseline anti-CCP status remains the best predictor of 5-year outcome

Individuals changing from anti-CCP negative to positive were more likely to have erosions and a high Larsen score at 5 years than were those who remained negative (Table 2). They were also more likely to have been treated with a DMARD, which, given that anti-CCP status was not known at the time of treatment, is likely to be a marker of disease severity. In contrast, individuals changing from anti-CCP positive to negative had radiographic and functional outcomes at 5 years similar to those that remained positive, although a trend towards lower disease activity appeared, as assessed by DAS28 and swollen-joint count (Table 2). Combined baseline and 5-year anti-CCP measurements did not improve the sensitivity or specificity for detection of radiographic joint damage or functional impairment (HAQ ≥ 1) compared with baseline anti-CCP status alone (Table 3). Clinical outcomes at 5 years differed in patients whose RF status changed between baseline and 5 years compared with those who remained consistently RF negative or RF positive (Additional file 1, Supplemental table S2). However, these differences were largely accounted for by differences in anti-CCP status at baseline. After adjustment for the presence of anti-CCP antibodies at baseline, no significant differences in 5-year outcomes were found between the patients who remained RF positive at 5 years and the 35% who converted from RF positive to negative (Additional file 1, Supplemental table S2).

**Table 3:** Sensitivity, specificity and likelihood ratios for presence of radiographic damage and functional disability at 5 years according to baseline and 5-year autoantibody status

Autoantibody status	Erosions				Larsen score > 15				HAQ ≥ 1			
	
	Sensitivity	Specificity	Accuracy	LR	Sensitivity	Specificity	Accuracy	LR	Sensitivity	Specificity	Accuracy	LR
**Anti**-**CCP^+^**												
Baseline	0.56	0.89	0.74	5.0	0.65	0.85	0.79	4.3	0.41	0.78	0.61	1.8
Year 5	0.56	0.89	0.75	5.1	0.66	0.86	0.79	4.5	0.41	0.78	0.61	1.8
Base or Year 5	0.59	0.88	0.75	5.1	0.69	0.85	0.79	4.5	0.43	0.77	0.62	1.8
**RF^+^**												
Baseline	0.44	0.84	0.67	2.8	0.50	0.82	0.71	2.7	0.36	0.77	0.59	1.6
Year 5	0.40	0.84	0.65	2.5	0.42	0.81	0.68	2.2	0.36	0.82	0.61	1.9
Base or Year 5	0.54	0.76	0.67	2.3	0.60	0.74	0.69	2.3	0.45	0.72	0.60	1.6

Logistic regression model; LR, likelihood ratio.

Failing to identify individuals who test autoantibody negative at baseline but later become seropositive is a potential clinical concern; therefore, the 399 individuals testing negative for both RF and anti-CCP at baseline were analyzed as a separate group. Only 1.5% of these patients converted to become anti-CCP positive between baseline and 5 years. More (6.7%) converted to become RF positive; however, most outcomes at 5 years were not significantly different in patients becoming autoantibody positive compared with those that remained autoantibody negative (Additional file 1, Supplemental table S3). The exception is that patients who became autoantibody positive by 5 years were more likely to have received a DMARD than were those remaining negative; this may reflect that those patients had other features of severe disease.

### Baseline anti-CCP titer predicts erosive joint damage at 5 years

A higher baseline anti-CCP antibody concentration among anti-CCP-negative individuals was the strongest predictive factor for conversion to anti-CCP positive in the first 5 years of disease (Table 2). We therefore examined the relation between baseline anti-CCP titer and radiographic and functional outcome. Baseline anti-CCP concentration was significantly associated with the presence and severity of erosions (Table 4). Classifying patients according to anti-CCP titer revealed a dose-response relation between increasing baseline anti-CCP titers and the presence and severity of erosions at 5 years (Table 5). Even baseline anti-CCP titers between 2 and 5 U/ml were highly significantly associated with radiologic outcome at 5 years. Consequently, reducing the cut-off threshold for anti-CCP titer to 2 U/ml improved the sensitivity for predicting erosions from 53% to 59%. However, the corresponding loss of specificity meant that the overall accuracy remained similar to the standard cut-off of 5 U/ml (Table 6).

**Table 4:** Logistic regression model exploring association between baseline anti-CCP titer and radiographic and functional outcome at 5 years

**5**-**year outcome**	OR (95% CI)	*P*	Adjusted OR^a ^	Adjusted *P*^a^
**Erosions**	1.03 (1.02, 1.04)	< 0.0001	1.03 (1.01, 1.03)	< 0.0001
**Larsen score > 15**	1.03 (1.02, 1.03)	< 0.0001	1.02 (1.01, 1.03)	< 0.0001
**HAQ ≥ 1**	1.01 (1.01, 1.02)	< 0.0001	1.00 (0.99, 1.01)	0.47

**^a^**Adjusted for age, gender, symptom duration and CRP, RF, HAQ, and swollen- and tender-joint counts at baseline. OR, odds ratio; CI, confidence interval. Baseline anti-CCP titer was available in 596 patients. ACR, American College of Rheumatology criteria for rheumatoid arthritis; DAS, disease activity score; HAQ, Health Assessment Questionnaire.

**Table 5:** Logistic regression for 5-year radiographic damage according to categories of baseline anti-CCP titer

**Anti**-**CCP titer**	Erosions					Larsen score ≥ 15				
	**No with erosions/total in group (%)**	**OR (95% CI)**	***P *value**	**Adjusted OR (95% CI)^a^**	***P *value^a^**	**No with Larsen ≥ 15/total in group (%)**	**OR (95% CI)**	***P *value**	**Adjusted OR (95% CI)^a^**	***P *value^a ^**

**≤ 2**	85/324 (26%)	Ref	-	Ref	-	47/323 (15%)	Ref	-	Ref	-
**> 2 to 5**	14/25 (56%)	3.6 (1.5, 8.3)	0.003	3.4 (1.2, 9.8)	0.02	11/25 (44%)	4.6 (1.9, 11)	0.0001	4.3 (1.4, 13)	0.009
**> 5 to 20**	16/22 (73%)	7.5 (2.7, 20)	< 0.0001	3.8 (1.3, 12)	0.02	14/22 (64%)	10.3 (3.9, 27)	< 0.0001	5.3 (1.7, 16)	0.003
**> 20**	94/116 (81%)	12.4 (6.6, 22)	< 0.0001	9.0 (4.4, 18)	< 0.0001	77/115 (67%)	11.9 (6.7, 21)	< 0.0001	9.7 (4.7, 20)	< 0.0001

**^a^**Adjusted for age, gender, symptom duration and CRP, RF, HAQ, and swollen- and tender-joint counts at baseline. CI, confidence interval; OR, odds ratio.

**Table 6:** Prediction of radiographic damage at 5 years for different cut-off levels of baseline anti-CCP titer

**Anti**-**CCP titer cut**-**off**	Erosions					Larsen score ≥ 15				
	
	Sensitivity	Specificity	Accuracy	LR+ve	**LR**-**ve**	Sensitivity	Specificity	Accuracy	LR+ve	**LR**-**ve**
**1**	0.80	0.54	0.65	1.7	0.4	0.88	0.52	0.63	1.8	0.2
**2**	0.59	0.86	0.74	4.2	0.5	0.69	0.82	0.78	3.8	0.4
**3**	0.56	0.89	0.75	5.0	0.5	0.66	0.85	0.79	4.4	0.4
**4**	0.54	0.90	0.74	5.3	0.5	0.62	0.86	0.79	4.6	0.4
**5**	0.53	0.90	0.74	5.2	0.5	0.61	0.86	0.79	4.5	0.5
**10**	0.50	0.91	0.73	5.5	0.6	0.57	0.87	0.78	4.5	0.5

LR+ve, positive likelihood ratio; LR-ve, negative likelihood ratio.

### Change in anti-CCP titer between baseline and 5 years does not correlate with clinical outcomes

A small but statistically significant increase was found in anti-CCP titer between baseline and 5 years in this cohort. The median absolute change was 0.11 (IQR, -0.5 to 0.77; *P *= 0.0007), which equated to a median percentage increase of 10% (IQR, -34% to 95%). Change in anti-CCP antibody titer was negatively correlated with the baseline titer (Spearman rho, -0.24 for absolute change and -0.4 for percentage change; *P *< 0.00001; that is, the higher the baseline anti-CCP titer, the less likely it was to increase). However, no correlation between change in anti-CCP titer and any of the markers of disease activity or severity at 5 years was detected. Changes in HAQ, DAS28, swollen and tender joint count and CRP between baseline and 5 years likewise did not correlate with change in anti-CCP concentration (data not shown). This remained true after adjustment for baseline anti-CCP concentration and DMARD treatment.

## Discussion

The key message of this study is that anti-CCP antibody status remains stable up to 5 years after disease onset in patients with IP. Patients switching from anti-CCP positive to negative retained the disease characteristics of persistently anti-CCP-positive patients. In contrast, 5-year radiographic outcomes were significantly worse in individuals converting from anti-CCP negative to positive, compared with those who remained negative; however, only 1.5% of patients negative for both anti-CCP and RF at baseline had converted to anti-CCP positive at 5 years. Repeating anti-CCP antibody measurement at 5 years added no additional predictive value in detection of radiographic damage or functional disability over baseline anti-CCP status alone.

Consistent with previous studies, RF status was more likely to change over time, with a lower proportion of subjects testing positive for RF at 5 years than at presentation [15,16]. However, baseline anti-CCP status was a strong predictor of change in RF status, and after accounting for the effect of anti-CCP, change in RF status in either direction was not significantly associated with clinical outcomes. This is in keeping with our previous findings in this cohort that anti-CCP antibodies predict more-severe disease irrespective of RF status [8,25].

Consistent with the known specificity of a positive anti-CCP status for RA and with its strong association with disease severity, patients with IP who will not satisfy the 1987 criteria for RA at any time during the first 5 years of follow-up were much less likely to be anti-CCP positive and displayed a much milder disease course. However, it is now impossible for practitioners to predict accurately at baseline which patient will prospectively go on to satisfy cumulatively the 1987 ACR criteria for RA within the 5 first years of follow-up. This should be, however, improved with the use of the 2010 ACR/EULAR criteria.

Baseline anti-CCP titer predicted both the presence and severity of erosive joint damage at 5 years in this cohort. A dose-response relation was observed, and individuals with a baseline anti-CCP concentration of more than 4 times the upper limit of normal were 10 times more likely to have a Larsen score above 15 (highest tertile) at 5 years as were those with a baseline anti-CCP titer of less than 2 (Table 5). Consistent with this, higher anti-CCP antibody levels have been found to associate with increased disease activity and radiographic progression in patients with RA [5,17,26]. However, a previous study investigating this relation in subjects with undifferentiated IP found no significant association between baseline anti-CCP concentration and DAS28, HAQ, or radiographic progression at 2 years [16]. The disparity with our results may relate to the short follow-up time in that study, as the strength of correlation between baseline anti-CCP titer and clinical indices has been shown to increase gradually in the first 5 years of disease in patients with early RA [17]. In support of our findings, higher anti-CCP titers predicted development of persistent arthritis in patients with IP [27]. Lowering the threshold for anti-CCP positivity marginally improved the predictive value for erosive disease in our cohort, but at the cost of loss of specificity, which is similar to the findings of Mjaavatten *et al. *[27] for prediction of persistent arthritis. Insufficient evidence exists to recommend altering the threshold for anti-CCP positivity; however, these findings highlight the importance of considering the absolute anti-CCP titer when evaluating the likely prognosis in patients with early IP [13,27].

In keeping with previous studies, we found no significant associations between change in anti-CCP titers and 5-year clinical outcomes [16,17]. It is not clear whether anti-CCP titers are affected by DMARD treatment, and study findings have been inconsistent [17-19]. We included an adjustment for ever having received a DMARD, which did not materially alter our findings; however, we cannot exclude that DMARD treatment may have influenced associations between change in anti-CCP concentrations and clinical outcomes. Nevertheless, available data suggest that any associations between variation in anti-CCP titer and disease severity are relatively weak and are unlikely to add significant prognostic information over that of baseline anti-CCP titer alone.

The proportion of patients positive for anti-CCP (30%) or RF (26%) at 5 years was much lower than is typically seen in cohorts with established RA, even though 73% of patients fulfilled ACR criteria for RA by 5 years. These percentages are similar to those observed in other early arthritis cohorts [14]. Our findings indicate that this discrepancy is unlikely to be due to a large proportion of subjects converting to autoantibody positivity in later disease. An alternative explanation may be that seronegative individuals are more likely to have milder disease and therefore are underrepresented in hospital-based cohorts with established RA.

Strengths of this study include the large sample size tested in the analysis, the availability of serum samples at baseline and 5 years, and the repeated testing of borderline samples to ensure the most accurate results. A potential weakness is the fact that no information was available regarding RF or anti-CCP status in the intervening years, so it is possible that "ever positive" status is a better predictor of outcome. However, given the stability observed for anti-CCP status over a 5-year period, it is unlikely to vary to a greater extent over the intervening years.

## Conclusions

Anti-CCP antibody status is unlikely to change over time in patients with early IP, and 5-year autoantibody testing adds no prognostic value over baseline measurement alone. Our findings, in conjunction with those of previous studies, provide no evidence of a benefit of routine retesting of anti-CCP antibodies or RF in patients with IP [15,16]. Repeated testing may nevertheless be useful in specific clinical scenarios, such as in patients negative for both RF and anti-CCP at presentation but with severe disease, in whom autoantibody status may influence treatment decisions, such as the use of Rituximab, which is less effective in seronegative patients. This study additionally demonstrates that higher anti-CCP titer at presentation in patients with IP predicts both development and severity of erosive disease, even at levels below the cut-off for anti-CCP positivity. Absolute anti-CCP titer should therefore be considered both a diagnostic and prognostic marker in patients with early IP [13,27].

## Abbreviations

ACPA: anti-citrullinated protein antibody; ACR: American College of Rheumatology; anti-CCP: anti-cyclic citrullinated peptide; ACPA: anti-citrullinated protein antibody; CRP: C-reactive protein; DAS28: Disease Activity Score (28 joints); DMARDs: disease-modifying antirheumatic drugs; EULAR: The European League Against Rheumatism; HAQ: Health Assessment Questionnaire; IP: inflammatory polyarthritis; IQR: interquartile range; LR: likelihood ratio; NOAR: Norfolk Arthritis Register; OR: odds ratio; RA: rheumatoid arthritis; RF: rheumatoid factor; ROC: receiver-operating characteristic; SE: shared epitope.

## Competing interests

The authors declare that they have no competing interests.

## Authors' contributions

MLB, SV, and MB performed the statistical analysis and drafted the manuscript. DP participated in the design of the study. DPS is the founder and principal investigator of the NOAR study. WT and AB conceived the study and participated in its design and coordination. All authors read and approved the manuscript for publication.

## Supplementary Material

Additional file 1 **Supplementary Tables S1 to S4**. Supplementary Tables S1 to S4. Click here for file
