# Supplementary material for: Long-term stability of anti-cyclic citrullinated peptide antibody status in patients with early inflammatory polyarthritis
Source: Arthritis Res Ther. 2012 May 9;14(3):R109. doi: 10.1186/ar3834 (PMC3446486; doi:10.1186/ar3834)
Supplement: Additional file 1 — Supplementary Tables S1 to S4. Supplementary Tables S1 to S4. [file ar3834-S1.DOC]

**Supplementary Table S**1: Baseline characteristics according to baseline and 5 year RF status

|  | **RF negative at baseline** | | | **RF positive at baseline** | | |
| --- | --- | --- | --- | --- | --- | --- |
| **5 year RF status** | **RF negative yr 5**  **(n=410, 90%)**  **RF -/-** | **RF positive yr 5**  **(n=48, 10%)**  **RF -/+** | **p value** | **RF negative yr 5**  **(n=64, 35%)**  **RF +/-** | **RF positive yr 5**  **(n=118, 65%)**  **RF +/+** | **p value** |
| **Baseline characteristic** |  |  |  |  |  |  |
| Age | 52.5 (40.8,63.1) | 51.4 (43.9,65.8) | 0.93 | 52 (43,63) | 56 (49,65) | 0.05 |
| Female | 273 (67%) | 34 (71%) | 0.55 | 43 (67%) | 69 (58%) | 0.25 |
| Symptom duration | 4.9 (2.2,10.4) | 7.5 (3.2,11.8) | 0.21 | 5.7 (2.6,11.7) | 4.7 (2.1,9.9) | 0.27 |
| Anti-CCP +ve | 38 (9%) | 21 (44%) | <0.0001 | 32 (50%) | 104 (88%) | <0.0001 |
| Anti-CCP titre | 0.9 (0.5,1.6) | 1.7 (1,30) | 0.0001 | 3.1 (0.8,59) | 50 (16,101) | 0.0001 |
| SE – 1 or 2 copies | 214 (56%) | 30 (70%) | 0.09 | 43 (72%) | 78 (71%) | 0.92 |
| HAQ | 0.6 (0.3,1.2) | 0.9 (0.3,1.4) | 0.06 | 0.6 (0.3,1.4) | 0.9 (0.4,1.6) | 0.23 |
| CRP | 3 (0,11) | 7 (1,17) | 0.11 | 8 (2,24) | 11 (3,23) | 0.26 |
| Swollen joints | 6(2,12) | 8 (4,15) | 0.12 | 8 (4,14) | 8 (3,18) | 0.52 |
| Tender joints | 7(2,15) | 8 (4,21) | 0.26 | 7 (3,16) | 10 (4,17) | 0.22 |
| DAS28 | 3.7 (2.8,4.8) | 4.6 (3.4, 5.2) | 0.01 | 3.7 (3.0,5.3) | 4.5 (3.3,5.4) | 0.17 |
| Current smoker | 92 (22%) | 11 (23%) | 0.94 | 13 (20%) | 44 (37.3%) | 0.02 |
| Fulfils ACR criteria | 156 (38%) | 20 (42%) | 0.63 | 45 (70%) | 92 (78%) | 0.25 |

Continuous variables expressed as median (interquartile range) and compared using Mann Whitney U test. Categorical variables expressed as number (percentage) and compared using Chi square test. RF= rheumatoid factor; CRP=C-reactive protein; ACR= American College of Rheumatology criteria for rheumatoid arthritis; HAQ = Health assessment questionnaire

**Supplementary Table S**2: 5 year outcome according to baseline and 5 year RF status adjusted for baseline anti-CCP status

|  | **RF negative at baseline** | | | | **RF positive at baseline** | | | |
| --- | --- | --- | --- | --- | --- | --- | --- | --- |
| **5 year outcome** | **RF negative yr5**  **(n=410, 90%)**  **RF -/-** | **RF positive yr5**  **(n=48, 10%)**  **RF -/+** | **p value** | **Adjusted p value** | **RF negative yr5**  **(n=64, 35%)**  **RF +/-** | **RF positive yr5**  **(n=118, 65%)**  **RF +/+** | **p value** | **Adjusted p value** |
| Erosions † | 106 (32%) | 23 (50%) | 0.02 | 0.9 | 33 (59%) | 70 (75%) | 0.04 | 0.88 |
| Larsen score ‡ | 3 (0,13) | 11 (0,28) | 0.04 | 0.72 | 6 (1,20) | 14 (1,40) | 0.34 | 0.28 |
| HAQ # | 0.6 (0.1,1.4) | 1.1 (0.5,1.9) | 0.015 | 0.02 | 0.9 (0.3,1.6) | 1.4 (0.5,2) | 0.08 | 0.11 |
| Swollen joints ‡ | 1 (0,3) | 2 (0,8) | 0.02 | 0.36 | 2 (0,4) | 4 (0,9) | 0.01 | 0.15 |
| Tender joints ‡ | 1 (0,6) | 4 (0,10) | 0.11 | 0.25 | 2 (0,6) | 3 (0,10) | 0.001 | 0.09 |
| DAS28 # | 2.3 (1.4,3.3) | 2.9 (2.0,4.5) | 0.02 | 0.41 | 2.7 (1.8,3.5) | 3.4 (2.2,4.2) | 0.017 | 0.42 |
| DMARD received † | 133 (33%) | 32 (67%) | <0.0001 | 0.02 | 40 (63%) | 96 (81%) | 0.006 | 0.77 |
| Fulfils ACR criteria† | 254 (62%) | 42 (88%) | 0.001 | 0.02 | 56 (88%) | 111 (94%) | 0.13 | 0.50 |

Continuous variables expressed as median (interquartile range) and compared using Mann Whitney U test. Categorical variables expressed as number (percentage) and compared using Chi square test. † Logistic regression used to compare significance between groups. ‡ Negative binomial regression used to compare significance between groups. # Median regression used to compare significance between groups.

**Supplementary Table S3: 5 year outcomes according to 5 year autoantibody status in 399 subjects negative for both RF and anti-CCP at baseline**

|  | **5 year autoantibody status** | | |
| --- | --- | --- | --- |
| **5 year outcome** | **Negative**  **(CCP -ve and RF –ve)**  **(n= 367, 92%)** | **Positive**  **(CCP +ve or RF +ve)**  **(n=32, 8%)** | **p value** |
| Erosions | 75 (26%) | 11 (38%) | 0.16 |
| Larsen score | 2 (0,9) | 3 (0,17) | 0.48 |
| HAQ | 0.5 (0, 1.4) | 0.9 (0, 1.7) | 0.48 |
| Swollen joints | 0 (0,2) | 1 (0,4) | 0.19 |
| Tender joints | 1 (0,6) | 4 (0,9) | 0.43 |
| DAS28 | 2.2 (1.3, 3.1) | 2.3 (1.6, 3.8) | 0.47 |
| DMARD received | 99 (27%) | 17 (53%) | 0.002 |

Continuous variables expressed as median (interquartile range) and compared using Mann Whitney U test. Categorical variables expressed as number (percentage) and compared using Fisher’s exact test.

**Supplementary Table S4: Baseline characteristics and 5 year outcome in patients with inflammatory polyarthritis who will never satisfy 1987 criteria within the first 5 years of follow-up, according to baseline serologic status**

| **Serologic status at baseline** | **Anti-CCP negative**  **(n=169, 95%)** | **Anti-CCP positive**  **(n=8, 5%)** | **p value** | **RF negative**  **(n=162, 92%)** | **RF positive**  **(n=15, 8%)** | **p value** |
| --- | --- | --- | --- | --- | --- | --- |
| **Baseline characteristic** |  |  |  |  |  |  |
| Age (n=177) | 47.3 (34.2, 56.5) | 59.4 (52.1, 63.7) | 0.05 | 47.9 (34.3, 57.9) | 46.8 (32.1, 53.7) | 0.48 |
| Female (n=177) | 111 (66%) | 3 (38%) | 0.10 | 106 (65%) | 8 (53%) | 0.35 |
| Symptom duration (n=177) | 4.5 (1.8, 9.1) | 4.5 (3.4, 7.5) | 0.83 | 4.5 (1.7, 9.2) | 4.2 (2.3, 6.6) | 0.78 |
| RF positive (n=177) | 12 (7%) | 3 (38%) | 2.6E-3 | - | - | - |
| Anti-CCP positive (n=177) | - | - | - | 5 (3.1%) | 3 (20%) | 2.6E-3 |
| Anti-CCP titre (n=170) | 0.8 (0.4, 1.3) | 11.1 (9.4; 46.5) | 7.7E-6 | 0.8 (0.5, 1.4) | 0.7 (0.4, 1.5) | 0.75 |
| SE – 1 or 2 copies (n =158) | 86 (57%) | 5 (63%) | 0.77 | 83 (58%) | 8 (57%) | 0.97 |
| HAQ (n=177) | 0.25 (0, 0.625) | 0.312 (0, 0.437) | 0.74 | 0.25 (0, 0.625) | 0.25 (0, 0.5) | 0.41 |
| CRP (n=165) | 2 (0, 6.5) | 6.4 (0.5, 9.0) | 0.28 | 2 (0, 6.5) | 3 (0, 14) | 0.13 |
| Swollen joints (n=177) | 2 (1,4) | 1 (0.5,1.5) | 0.06 | 2 (1, 4) | 1 (0, 2) | 0.02 |
| Tender joints (n=177) | 3 (1,7) | 6.5 (2.5, 7.5) | 0.20 | 4 (1, 8) | 2 (1, 3) | 0.09 |
| DAS28 (n=165) | 2.8 (2.0, 3.6) | 3.0 (2.3, 3.5) | 0.63 | 2.8 (2.1, 3.6) | 2.2 (2.0, 2.8) | 0.07 |
| Current smoker (n=177) | 44 (26%) | 2 (25%) | 0.95 | 42 (26%) | 4 (27%) | 0.95 |
| **Markers / outcome year 5** |  |  |  |  |  |  |
| RF positive (n=177) | 9 (5%) | 4 (50%) | 2.2E-6 | 6 (3.7%) | 7 (47%) | 1.0E-9 |
| Anti-CCP positive (n=177) | 1 (0.6%) | 7 (88%) | 6.4E-31 | 5 (3%) | 3 (20%) | 2.6E-3 |
| Anti-CCP titre (n=177) | 1.1 (0.6, 1.5) | 25.7 (8.1, 82.1) | 1.9E-6 | 1.1 (0.7, 1.6) | 1.3 (0.7, 1.8) | 0.18 |
| Erosions (n=136) | 11 (8%) | 1 (17%) | 0.49 | 10 (8%) | 2 (17%) | 0.32 |
| Larsen score (n=136) | 1 (0, 4) | 0 (0, 20) | 0.84 | 1 (0, 4) | 1 (0, 3.5) | 0.80 |
| HAQ (n=176) | 0.25 (0, 0.625) | 0.06 (0, 0.56) | 0.59 | 0.25 (0, 0.625) | 0 (0, 0.625) | 0.46 |
| Swollen joints (n=124) | 0 (0, 1) | 0 (0, 0) | 0.09 | 0 (0, 1) | 0 (0, 1) | 0.81 |
| Tender joints (n=124) | 0 (0, 2) | 0 (0, 0) | 0.16 | 0 (0, 2) | 0 (0, 1) | 0.35 |
| DAS28 (n=120) | 2.0 (1.2, 2.4) | 1.2 (1.2, 1.8) | 0.19 | 1.9 (1.2, 2.4) | 2.1 (2.0, 2.1) | 0.44 |
| DMARD received (n=176) | 19 (11%) | 4 (50%) | 1.5E-3 | 16 (10%) | 7 (47%) | 5.4E-5 |

Continuous variables expressed as median (interquartile range) and compared using Mann Whitney U test. Categorical variables expressed as number (percentage) and compared using Chi square test. Number of patients available with non missing data are shown in parentheses. RF= rheumatoid factor; CRP=C-reactive protein; ACR= American College of Rheumatology criteria for rheumatoid arthritis; DAS28 = Disease activity score in 28 joints; HAQ = Health assessment questionnaire; DMARD = disease modifying anti-rheumatic drug.
